# Effects of Fruit and Vegetable-Based Nutraceutical on Cognitive Function in a Healthy Population: Placebo-Controlled, Double-Blind, and Randomized Clinical Trial

**DOI:** 10.3390/antiox10010116

**Published:** 2021-01-15

**Authors:** Juan Ángel Carrillo, Raúl Arcusa, María Pilar Zafrilla, Javier Marhuenda

**Affiliations:** Faculty of Health Sciences, Universidad Católica de San Antonio, 30107 Murcia, Spain; jacarrillo4@alu.ucam.edu (J.Á.C.); rarcusa@ucam.edu (R.A.); jmarhuenda@ucam.edu (J.M.)

**Keywords:** cognition, polyphenols, executive function, memory, attention, Stroop, TESEN, RIST

## Abstract

There is scientific evidence of the positive effect of polyphenols from plant foods on cognition, but not enough is known about the synergistic effect when multiple polyphenols are consumed and even less in a healthy non-elderly population. The aim of the present study is to investigate the possible effects of improvements in cognitive function in healthy people as a preparation based on micronized fruit and vegetables consumed. One hundred and eight subjects were selected, stratified by sex in the control intervention group (*n* = 53) and placebo (*n* = 55). Volunteers completed the study after two 16-week periods of consumption with a 4-week wash period between each phase. At the beginning and the end of each phase, volunteers performed the Stroop, TESEN, and RIST tests for the measurement of different cognitive function patterns. The results revealed statistically significant differences in all the variables of the tests carried out, especially compared with the placebo. Specially, the results obtained in the Stroop and TESEN test, in addition to the processing speed even with semantic interferences, were markedly better after the treatment with the product under study. Moreover, the consumption of the product under study clearly improves short-term memory, verbal and non-verbal, according to the results obtained in the RIST test. The results showed an improvement in executive function in terms of short-term memory, working memory, selective and sustained attention, and speed of processing.

## 1. Introduction

Polyphenols, including phenolic acids, flavonoids, such as anthocyanins or flavonols and tannins, vary depending on the fruits or vegetables where they are present: berries, onions, apples, parsley, celery, broccoli... [1,2]. They have a considerable capacity to neutralize free radicals and exert anti-inflammatory and neuroprotective effects [3,4] and are considered important in cognitive function. Although the mechanisms of action have not yet been clarified, these compounds are known to modulate cerebral blood flow, inducing changes in memory processing [5,6], improving neuronal connectivity and neuronal growth in the hippocampus [7], and synaptic plasticity [8] related to variations in nitric oxide (NO) levels [9].

The effect of bioactive compounds also depends on consumption and frequency levels [10,11] since there is evidence of the effects in acute consumption [12], but not so much in prolonged consumption over time. The brain is especially sensitive and prone to oxidative damage and the accumulation of reactive oxygen species (ROS) due to increases in oxygen consumption [13]. Polyphenol supplementation decreases the vulnerability of elderly people who present higher risk factors to oxidative stress, improving neuronal communicability [14,15].

There are authors who have investigated the synergistic effects of various plant compounds [16,17], but their effects have not been clearly demonstrated and more randomized trials are needed [12]. Some authors have shown that the consumption of a Mediterranean diet in young people leads to a decreased risk of dementia when they are elderly [18,19], and that in these healthy young subjects improved mood and a decreased risk of depression has been noted [20]. Other authors have demonstrated an association between a diet that is high in fruit and vegetables with improvements in executive functions [21].

Cognitive functions can be evaluated through cognitive tests in combination with other interventions as functional MRI [22]. These tests are agreed by the scientific community and are used as evidence for certain healthy statements [23,24]. In order to reach an adequate consumption of polyphenols, nutraceuticals can provide an easy means of consuming an adequate allowance of polyphenols [25,26,27], even though information on intervention studies in humans in clinical conditions is lacking [28,29,30].

Fruit and vegetables must be precursors to a new generation of treatments of diseases through diet and lifestyle [31], by the consumption of nutraceuticals [32,33]. However, few randomized studies on the effects of polyphenols on memory and learning have been conducted in human models in non-elderly populations [34], concluding positive effects on this benefits in animal models with the use of foods rich in flavonoids, evaluating memory dosing blueberry [35], memory with green tea [36], and learning with gingko biloba [37]. The objective of the present clinical trial is to evaluate the efficacy of continuous consumption of an extract based on multiple fruits and vegetables versus placebo in the improvement of cognitive function in healthy volunteers on several aspects of executive function, memory, and attention.

## 2. Materials and Methods

### 2.1. Experimental Design

**Figure 1:**
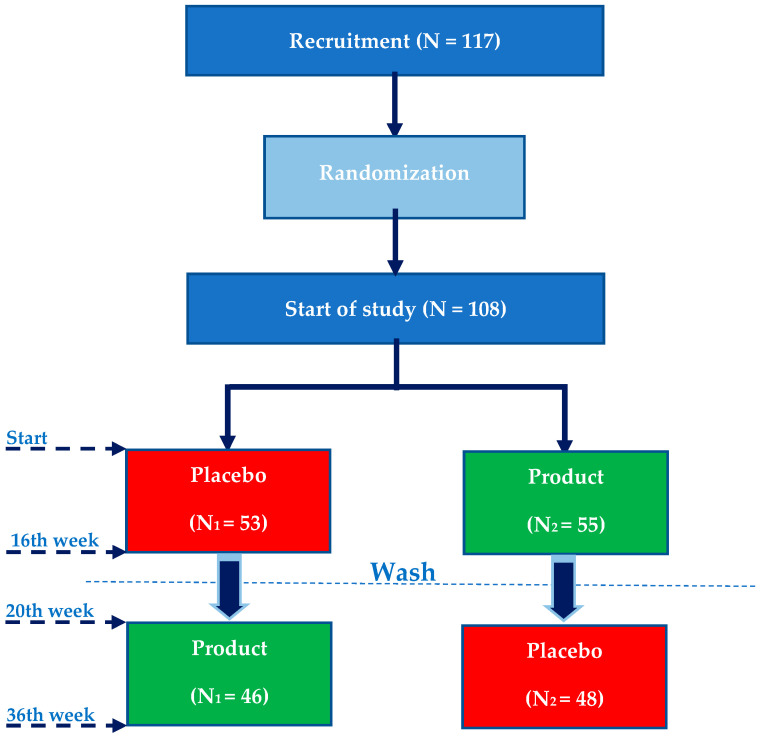
Phases of the intervention study. N1 = group 1 randomly selected. N2 = group 2 randomly selected.

The present intervention study is a randomized, crossover, double-blind, sex-stratified, placebo-controlled clinical control trial to assess the effect of daily intake of an encapsulated fruit, vegetable, and berry juice powder concentrate (Juice Plus+ Premium^®^, The Juice Plus Company^®^, Collierville, TN, USA) on cognitive functions. The duration of consumption was two periods of 16 weeks with an intermediate washout period of 4 weeks, and a daily intake of one of the products randomly assigned (product or placebo) (Figure 1). Once the first period was over and after the 4-week washout period, the groups were crossed so that all participants consumed both the product and the placebo.

During the experimental phase, each subject made a total of four visits throughout the research period to performing the relevant cognitive tests (Table 1). The protocol of this research study was approved by the ethics committee of the Catholic University of Murcia (date: 24 November 2017; Code: CE111702). The study was carried out following the Standards of Good Clinical Practice and the conditions that should govern human research studies that are defined in the Declaration of Helsinki. Current European legislation on the protection of personal data was complied with (Regulation (EU) 2016/679).

### 2.2. Study Population

A total of 117 volunteers were selected by means of distribution by mail and posters among students and university staff to determine their participation in the study. Of them, 108 fulfilled the inclusion criteria. 16 volunteers were rejected since they no longer met the selected inclusion/exclusion criteria or refused to continue. The total number of subjects included in the study was 92. Of these, 47 were male (51.09%) and 45 were women (48.91%). The mean age was 32.74 ± years.

### 2.3. Inclusion and Exclusion Criteria

The volunteers had to meet the following inclusion criteria: signed informed consent; body mass index ≥18.5 and ≤35 Kg/m²; not suffer from chronic illness; not consume more than three pieces of fruit or vegetables a day; and be between 18 and 65 years of age. The presence of at least one of the following criteria was a reason for exclusion from the clinical trial: being under medical or pharmacological treatment; having an allergy to fruit and/or vegetables; being on a diet; vegetarian or vegan; being a smoker; consuming more than 3 glasses of alcohol (wine or beer) a day; pregnancy; having undergone major surgery in the last 3 months; irregular or insufficient sleep; having donated more than 0.5 L of blood in the last month. To verify compliance with these requirements, the volunteers were interviewed, weighed and measured, and they filled out a questionnaire on lifestyle and nutrition habits used in previous studies [38] and by other authors [39].

### 2.4. Tested Product

The product tested contained a homogenized mixture of dehydrated juice and pulps of grapes and berries, fruits, and vegetables (56%) in variable proportions of apple, carrot, concord grape, pomegranate, orange, pineapple, blueberry, lingonberry, bilberry American, blackberry, cabbage, garlic, myrtle, citrus, mango, raspberry, acerola, peach, date, parsley, broccoli, spinach, kale, tomato, elderberry, blackcurrant, plum, beet, coating agent (pullulan), cocoa powder, green tea extract, tocopherol blend, ginger root powder, calcium ascorbate, artichoke leaf extract, grape seed extract, rice bran, spirulina powder, anti-caking agent: silicon dioxide, magnesium salts of fatty acids, citrus extract, lutein, beta-carotene, lycopene, astaxanthin and maltodextrin. The mixture was encapsulated in a white pullulan capsule. Each package contained sufficient capsules for 4 months of consumption (each period of consumption) considering a dose of 6 capsules a day (3 in the morning with breakfast and 3 in the evening with dinner).

The polyphenolic characterization was performed with UHPLC-QqQ-MS, in a previous study in 2017 by Bresciani and col. The product showed a total of 119 polyphenolic compounds belonging to different phenolic families: flavonols, such as kaempferol and quercetin, anthocyanins, and flavones [40]. The daily consumption of 6 capsules used in this study is the usual consumption in other intervention studies that have been performed previously involving a total of 600 mg of phenolic mixture [41,42,43]. The placebo was made of microcrystalline cellulose and had the same appearance and dosage as the product in order to avoid differentiation by the subjects or the research staff.

### 2.5. Cognitive Tests

To evaluate selective attention, the Stroop test was used [44]. This test has been used in studies in adults [45,46] and even in children [47] and is widely used in clinical studies to evaluate the effects of flavonoids on memory and attention both in children and adults [48]. Volunteers have to read as many items as possible from three independent consecutive parts resulting in three scores: P scores: number of words read on the page to read words, in which different colors appear written in one ink, C scores: number of items made on the name colors page, in which words written in different colors appear and the volunteers have to name the ink of the color with which they are written and PC scores: number of items made on the page of words-colors, in which you have to name the color of the ink with which the word is written, each word being a different color. The difficulty increases in each part, as there is semantic interference. Two methods are used for outcome measures: the time that the subject takes to complete 100 elements, and the number of elements completed in 45 s.

Moreover, the TESEN test used in the present study (Test of the trail) was developed in Spain for the evaluation of executive functions and working memory and it is suitable for intervention studies with a healthy population [49]. It is an individual application test to assess the executive functioning of youth and adults using a visualmotor planning task. It permits evaluation of planning capacity, working memory, mental flexibility, alternation, sustained attention, prospective memory, the speed of perceptual processing and the fluidity of motor response.

It is made up of four tests (trails) of increasing difficulty. In the first, the subject must join the numbered points in ascending order. In the second, subjects must do the same process but in descending order. In the third path, the subject must join the numbers in increasing order alternating the color. In the fourth path, it is treated as in the case of the third but alternating geometric figures.

The test consists of three scores for each of the trails: Speed score (S): reflects the ability to solve tasks that require increasing attention and executive control; accuracy score or precision (P): reflects the number of hits and misses that the subject makes during the completion of each trail; and execution score (E): reflects the efficiency with which the evaluated person has carried out the task, taking into account speed and precision.

As a memory evaluation method, the RIST test (short adaptation of the RIAS test) [50], used in young people and children as an evaluation of working memory, was also performed in this study [51]. It consists of two tests: riddles and categories. The direct scores obtained are transferred to typical scores (TS) according to the scale table. The TSs are added up and the RIST index (RI) is calculated.

### 2.6. Statistic Analysis

The Kolmogorov–Smirnov test was used to check for normal distribution of continuous data. This way, continuous variables were presented as mean ± standard deviation (SD) in the baseline conditions and in their evolution. This analysis was performed for the total group of individuals who participated in the study in both the placebo and product consumption periods. The homogeneity of the population at baseline with respect to demographic variables, medical history and other clinical parameters were also analyzed. For the quantitative variables, *t*-Student comparisons were developed between the two branches of the study.

To analyze the differences between the groups (experimental and control) in the evolution of the different variables, an analysis of variance was performed for repeated measures with time as an intra-subject factor and product as an inter-subject factor. This way, differences in each analyzed variable were established, taking these factors into account. The Bonferroni test was performed for post-hoc analysis. Significant effects (with the option of assuming equality of variances or not) were compared. In the set of statistical tests, the significance level used was 0.05. Statistical analysis was carried out with SPSS 24 computer software (SPSS, Inc., Chicago, IL, USA).

## 3. Results

### 3.1. Stroop Test

Table 2 shows the results along with the standard deviations of the number of words read (W), the number of elements made on the name of colors page (C), and the combination of words and colors (WC) in the initial and finals times for placebo and product. The results obtained in the three variables showed values above 30 in the typical scores (TS) in the initial times, with normal values for executive functions and attentional cognitive flexibility and with no neurological damage in any of the volunteers who underwent the tests. It was verified that there were differences in the three variables and that the subjects were able to read 4.2 more words in the first reading words test, to read 3.56 more words when it was about reading colors and also when there was semantic interference in the WC variable (4.67).

**Figure 2:**
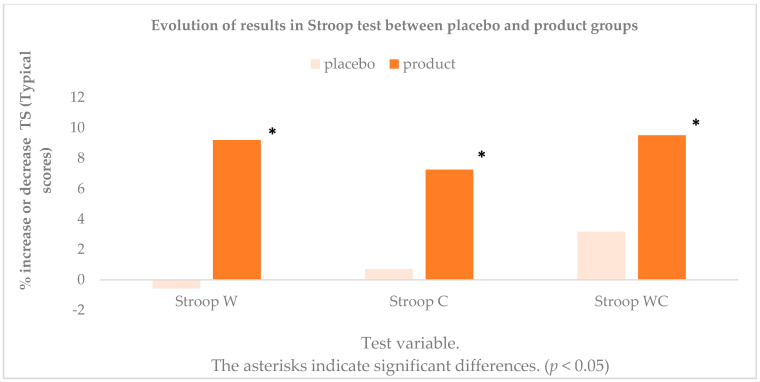
Evolution of results in the different variables of Stroop test in percentage increase or decrease. * indicate significant differences. (*p* < 0.05).

These differences were statistically significant (*p* < 0.01 for W, C and for WC) and were not so in consumption of the placebo (*p* = 0.783 for W, *p* = 0.744 for C and for *p* = 0.166 WC). When comparing by sex, there were no differences in any of the tests carried out. The results obtained in the different variables of the Stroop test showed a clear improvement in the speed of processing and in the attention span of the subjects, comparing the product and the placebo intake (Figure 2).

### 3.2. TESEN Test

As observed in the Stroop test, in the three variables measured in the TESEN test (trail test), significant differences were obtained in speed of execution (S), precision in terms of number of hits and misses (P), and execution in relation to speed and precision in the execution (E) between the final and initial phases of consumption of product (*p* < 0.01), but not for the placebo (*p* > 0.05), as shown in Table 3.

**Figure 3:**
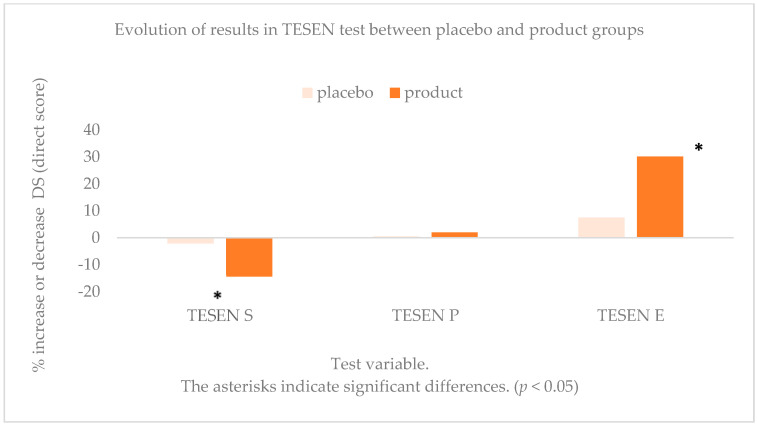
Evolution of results in the different variables of TESEN test in percentage increase or decrease. * indicate significant differences. (*p* < 0.05).

The decrease in time in variable S showed a very marked decrease (51 s) when the product was consumed (*p* < 0.01) compared with the placebo (*p* = 0.25). In the case of variable P, the increase was 1.96 points of direct score (DS) after the consumption of the product (*p* < 0.01) versus the placebo (*p* = 0.68). There were no differences between sexes in any of the tests carried out. The scores obtained in all the variables of the tests performed improved when compared with the consumption of the placebo. The differences are statistically significant and, in some cases, very marked (Figure 3).

### 3.3. RIST Test

**Figure 4:**
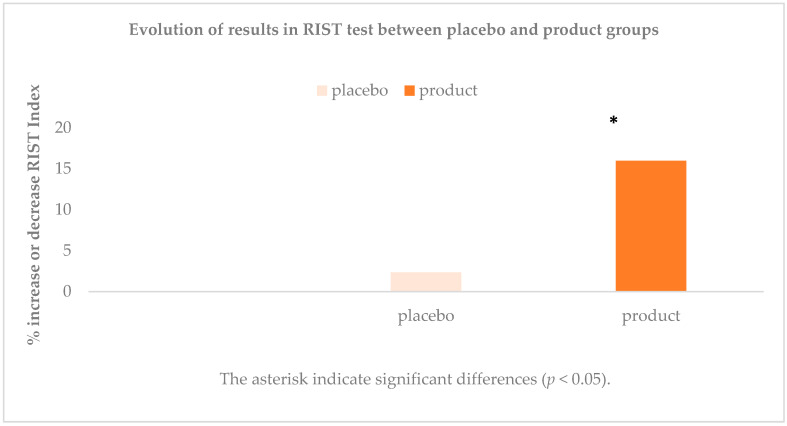
Evolution of results in RIST test between placebo and product groups in percentage increase or decrease. * indicate significant differences. (*p* < 0.05).

When comparing the results obtained in the scores of the RIST test, it was observed that there were differences between the initial and final phases in terms of product consumption. These differences were statistically significant (*p* < 0.01) but not so after placebo consumption (*p* = 0.30). Furthermore, in the case of product consumption, the differences between the final and initial phases were quite marked (15.27 points of difference in RI). When making the comparison differentiated by sex, there were no differences. The results are expressed in Table 4. As shown in Figure 4, the scores obtained in the RIST test improved when compared with the use of the placebo and there are statistically significant differences in RI.

## 4. Discussion

The purpose of the present study was to evaluate the efficacy of a fruit and vegetable-based extract versus placebo on cognitive function in healthy subjects in non-acute consumption. We hypothesized that an improvement could be achieved in accordance with previous studies although they were carried out in a population with neurodegenerative diseases or the polyphenolic extract used was not composed of a mixture of phenolic compounds [11,52,53,54,55,56]. The results obtained by other authors with different extracts followed a similar trend [46,57,58], either using melon concentrate supplementation and its influence on psychological stress and mental fatigue, using the administration of soy isoflavones in elderly men evaluating the visual-spatial memory and verbal fluency in the subjects, or in the evaluation of the efficacy of Ginkgo biloba extract in people over 55 years of age, where improvements were obtained assessing the speed of processing skills. On the other hand, people with a diet pattern high in fruits and vegetables showed better cognitive results in the Stroop test compared with those with a less healthy diet pattern, 5 and 25 years later, as described in the results of a cohort study published in 2015 [40].

Other authors have evaluated executive functions using the Stroop test combined with diet and physical activity in patients with metabolic syndrome. In this case, this combination causes an improvement in executive function [59]. In the present study, the observed decrease of more than 50 s on average in the execution speed (S) of the trails in the product consumption period indicated a clear improvement in perceptual processing speeds and in fluidity in visual-motor response. The precision and efficiency in the achievement of the trails, with fewer errors, also indicated an improvement in executive functions such as the subject’s capacity to plan, alternate and, ultimately, in the subject’s working memory.

The results obtained with the TESEN test was to those reported by other authors previously in interventional studies with the execution of the trail making test (TMT), in people with mild cognitive impairment, showing improvement in cognitive functions [60]. In these studies, as in one carried out with the Norwegian elderly population, the consumption of several foods rich in flavonoids demonstrated not only an improvement in test scores, but also that this improvement was dependent on the dose and type of flavonoid [61]. The most pronounced cognitive improvements were reported for carrots, citrus, and crucifers.

In the case of the RIST test, the results of more than 15 points of difference in the RI when the product is consumed also coincide with the results obtained in the TESEN test in terms of working memory and with those obtained by other authors in similar intervention studies, using the RIST test in a danish cohort study with children [51], evaluation with RVIP test in the administration of an extract rich in polyphenols [12] concluding that its consumption can be an alternative for the improvement of working memory and attention and in other interventions using similar cognitive tests [17,62].

The results obtained in the different variables of the three tests used in this study, which show an improvement in cognitive functions, may be due to the role played by polyphenols in synaptic plasticity through the CREB protein and modulating pathways of signaling and transcription factors [13,63,64]. On the other hand, flavonoids especially anthocyanins, have a positive effect on the brain cells associated with memory and neuronal function, mainly due to the increase of cerebral blood flow [6,65,66,67].

## 5. Conclusions

Our data indicate that chronic consumption of a polyphenolic extract from fruits and vegetables over 4 months improves the processes involved in executive functions such as working memory (ability to plan, alternation and fluidity of the motor response) and short-term memory. All the variables of the Stroop and TESEN test have been significantly higher than those compared with placebo, in addition to the processing speed even when semantic interferences (WC) are present. On the other hand, according to the results obtained in the RIST test, consumption of the product clearly improves short-term memory, whether this is verbal or non-verbal. The product under study may represent an alternative system in the prevention of normal cognitive deterioration caused by age, through the consumption of fruit and vegetables, in addition to an improvement in cognitive functions (immediate and working memory, sustained, and selective attention).

## Figures and Tables

**Table 1:** Follow-up actions at each unit visit.

Monitoring	VS	V1	V2	V3	V4
Informed Consent	X				
Inclusion/exclusion criteria	X				
History, lifestyle, dietary and eating habits	X				
Randomization	X				
Product/placebo delivery		X		X	
performing cognitive tests		X	X	X	X

V = Visits to the research unit. VS = visit start. X = action completed at each unit visit.

**Table 2:** Evolution of results obtained over time in Stroop test with product and placebo.

	Stroop Test W	Stroop Test C	Stroop Test WC
IPP ^1^	46.12 ± 7.60	49.39 ± 8.66	49.40 ± 8.68
FPP ^2^	45.86 ± 6.06	49.74 ± 6.03	50.96 ± 6.61
IPPD ^3^	45.71 ± 7.81	49.07 ± 8.63	49.11 ± 8.57
FPPD ^4^	49.91 ± 7.05 *	52.63 ± 9.41 *	53.78 ± 7.79 *

^1^ IPP: Initial phase placebo. ^2^ FPP: final phase placebo. ^3^ IPPD: initial phase product. ^4^ FPPD: final phase product. Results expressed in typical score (TS), mean and standard error of mean. * There are statistically significant differences (*p* < 0.05) between IPPD and FPPD phases.

**Table 3:** Evolution of results obtained over time in TESEN test with product and placebo.

	TESEN Test S	TESEN Test P	TESEN Test E
IPP ^1^	402.28 ± 53.32	97.74 ± 6.27	19.88 ± 4.18
FPP ^2^	393.85 ± 42.77	98.25 ± 9.03	21.38 ± 2.28
IPPD ^3^	406.83 ± 54.05	97.26 ± 6.43	19.69 ± 4.10
FPPD ^4^	355.68 ± 88.92 *	99.22 ± 1.55 *	25.62 ± 5.86 *

^1^ IPP: initial phase placebo. ^2^ FPP: final phase placebo. ^3^ IPPD: initial phase product. ^4^ FPPD: final phase product. Results expressed in direct scores (DS), mean and standard error of mean. * There are statistically significant differences (*p* < 0.05) between IPPD and FPPD phases.

**Table 4:** Evolution of results obtained over time in RIST test with product and placebo.

	RIST Test
IPP ^1^	95.91 ± 16.24
FPP ^2^	98.19 ± 13.82
IPPD ^3^	95.59 ± 16.35
FPPD ^4^	110.86 ± 10.01 *

^1^ IPP: initial phase placebo. ^2^ FPP: final phase placebo. ^3^ IPPD: initial phase product. ^4^ FPPD: final phase product. Results expressed in RIST Index (RI), mean and standard error of mean. * There are statistically significant differences (*p* < 0.05) between IPPD and FPPD phases.

## Abbreviations

The following abbreviations are used in this manuscript.

NO	nitric oxide
ROS	reactive oxygen species
MRI	magnetic resonance imaging
RIAS	Reynolds Intellectual Assessment Scales
RIST	Reynolds Intellectual Screening Test
TS	typical scores
RI	RIST Index
SD	standard deviation
TMT	trail making test
RVIP	Rapid Visual Information Processing
CREB	cAMP response element-binding
